# Diagnostic accuracy of ^18^F-FP-CIT PET for clinically uncertain Parkinsonian syndrome

**DOI:** 10.1038/s41598-023-42135-9

**Published:** 2023-09-12

**Authors:** Minyoung Oh, Seung Jun Oh, Sang Ju Lee, Jungsu S. Oh, Sun Ju Chung, Jae Seung Kim

**Affiliations:** 1 Department of Nuclear Medicine, Asan Medical Center, University of Ulsan College of Medicine, 88, Olympic-Ro 43-Gil, Songpa-Gu, Seoul, 05505 Korea; 2 Department of Neurology, Asan Medical Center, University of Ulsan College of Medicine, Seoul, Korea

**Keywords:** Biomarkers, Diseases, Neurology

## Abstract

^18^F-FP-CIT is a high-resolution imaging marker of nigrostriatal neuronal integrity, differentiating Parkinsonism with loss of dopaminergic terminals (presynaptic Parkinsonian syndrome [PS]) from Parkinsonism without nigrostriatal degeneration (non-PS). We assessed the diagnostic accuracy of ^18^F-FP-CIT PET in patients with clinically uncertain PS (CUPS) at the first visit. Among the 272 patients who underwent ^18^F-FP-CIT PET imaging at the first visit between September 2008 and July 2012, 111 had CUPS (age, 62.6 ± 10.5 y; male:female, 45:66; symptom duration, 13.1 ± 8.8 months). Uncertainty criteria included only one of the three cardinal signs of Parkinsonism, two signs without bradykinesia, or atypical signs. The baseline clinical and ^18^F-FP-CIT PET imaging diagnostic accuracy was compared with the accuracy of clinical diagnosis after > 2-year follow-up. Nuclear medicine physicians assessed the ^18^F-FP-CIT PET images visually. Focal dopamine transporter binding deficit in the posterior putamen was considered PS. Bilateral symmetric striatum without focal deficit, suggesting normal ^18^F-FP-CIT PET, and focal deficits elsewhere in the striatum suggesting vascular Parkinsonism were considered non-PS. Seventy-nine patients had PS, and 32 did not. Baseline clinical diagnosis included PS in 45 patients, non-PS in 24, and inconclusive in 42. Among patients in whom initial clinical diagnosis (PS or non-PS) was possible, the sensitivity, specificity, and accuracy of the baseline clinical and ^18^F-FP-CIT PET imaging diagnoses were 54.4, 50.0, and 53.2%, and 98.7, 100, and 99.1%, respectively. The respective positive and negative predictive values were 95.6 and 66.7%, and 100 and 97.0%. Among those with initially inconclusive diagnosis, 64.2% were eventually diagnosed with PS while 35.7% were diagnosed with non-PS. The final clinical diagnosis of these patients all matched those made by ^18^F-FP-CIT PET imaging, except in one patient with scan without evidence of dopaminergic deficit (SWEDD). ^18^F-FP-CIT PET diagnosis was more accurate than clinical diagnosis, reducing the false-negative and inconclusive clinical diagnosis rates at baseline in patients with CUPS.

## Introduction

Parkinsonian syndrome is a group of diseases characterized by signs of Parkinsonism, including bradykinesia, rigidity, tremor, and postural instability^1^. Idiopathic Parkinson’s disease (IPD) is the most common cause of Parkinsonism, but Parkinsonism has several other etiologies as well. It could be present in all alpha synucleinopathies, including Lewy body diseases, IPD, and multiple system atrophy^2^, and tauopathies, including corticobasal degeneration and progressive supranuclear palsy, which were defined as atypical Parkinsonisms^3,4^. The differential diagnoses of neurodegenerative Parkinsonian syndrome include essential tremor (ET), vascular Parkinsonism, drug-induced Parkinsonism, and psychogenic Parkinsonism^5^. ET mainly affects voluntary movements rather than rest. Resting tremor, cogwheel rigidity, and other Parkinsonian characteristics can be present in a subgroup of patients with ET, making the clinical diagnosis a challenge^6^.

Dopamine is a neurotransmitter with a vital role in movement regulation in the brain. Therefore, disruption of nigrostriatal pathway leads to a loss of dopamine and cause Parkinsonism. For example, IPD is characterized by the progressive loss of dopaminergic neurons in the substantia nigra pars compacta, leading to dopamine depletion in the striatum^7^. The dopamine transporter (DAT) controls the duration and intensity of dopaminergic neurotransmission by reuptake of dopamine into presynaptic terminals^8^ and is used in the imaging of presynaptic dopaminergic neuronal distribution for presynaptic Parkinsonian syndrome (PS) diagnosis.

Several DAT tracers for SPECT and PET, including ^123^I-FP-CIT, ^123^I-β-CIT, ^99m^Tc-TRODAT-1, and ^18^F-FP-CIT, are commercially available in the USA, Europe, Japan, Taiwan, and Korea^5,9^. Due to its high resolution and favorable kinetics, ^18^F-FP-CIT PET could achieve high accuracy in diagnosing Parkinsonism and in Parkinsonism differential diagnosis^10–12^.

This accuracy could help diagnose IPD in patients with clinically uncertain Parkinsonian syndrome (CUPS) when the clinical diagnosis is challenging due to the unclear characteristic symptoms of IPD that lead to some inconclusive diagnoses. This study investigated the diagnostic accuracy of ^18^F-FP-CIT PET in patients with CUPS when clinical diagnosis proves challenging.

## Materials and methods

### Subjects

We retrospectively selected patients with CUPS who underwent ^18^F-FP-CIT PET imaging as initial work-up for Parkinsonism during their first visit between September 2008 and July 2012 at our medical center. Uncertainty criteria included only one of the three cardinal signs of Parkinsonism, two signs without bradykinesia, or atypical signs^13^. Symptom duration was under three years, and follow-up was over two years in all patients by movement specialist. Patients with known causes of tremors such as hyperthyroidism, or with significant cognitive impairment with a minimal mental status score of ≤ 24 were excluded. The Institutional Review Board (IRB) of Asan Medical Center approved this study and informed consent was waived because of the retrospective nature of this study (IRB no. 2014–0688).

### Radiopharmaceutical synthesis

^18^F-FP-CIT was synthesized using a protic solvent (t-butanol or t-amyl alcohol) as a reaction solvent and N-[3′-(tosyloxy)propyl]-2β-carbomethoxy-3β-(4′-iodophenyl)nortropane as a precursor^14^. The decay-corrected radiochemical yield was 42.5% ± 10.9%; the radiochemical purity after purification with high-performance liquid chromatography was > 98%; the specific activity at the end of synthesis was 64.4 ± 4.5 GBq/μmol.

### ^18^F-FP-CIT PET/CT imaging

All ^18^F-FP-CIT PET images were acquired for ten minutes after three hours after intravenous administration of 185 MBq of ^18^F-FP-CIT using a Biograph Truepoint 40 PET/CT scanner (Siemens Medical Systems, USA). The medications that may influence DAT binding including benzatropine, D-amphetamine, methylphenidate were stopped before the ^18^F-FP-CIT PET/CT scanning. PET images were achieved in a 3-dimensional mode immediately after performing a brain computed tomographic scan for image fusion and attenuation correction. PET images were reconstructed with a TrueX algorithm and an all-pass filter using a 336 × 336 matrix.

### Visual analysis of ^18^F-FP-CIT PET/CT

**Figure 1:**
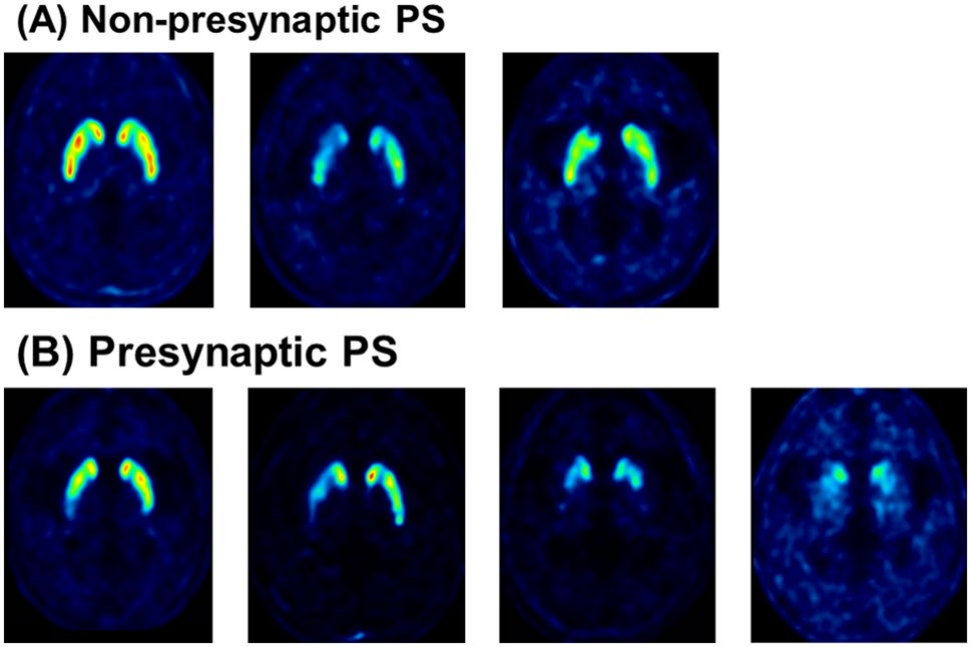
Visual interpretation criteria for (**A**) non-presynaptic Parkinsonian syndrome (non-PS) and (**B**) presynaptic Parkinsonian syndrome (PS). Patients with non-PS showed no significant DAT loss in the striatum or focal DAT loss in areas other than the posterior putamen. Patients with PS showed DAT loss in unilateral posterior putamen, unilateral putamen, bilateral putamen, or putamen and caudate nuclei.

All PET images were visually assessed by two board-certified nuclear medicine physicians with 15 and two years of clinical experience in nuclear medicine who were blinded to all clinical and diagnostic information (masked for review) working in consensus. ^18^F-FP-CIT PET image visual assessment was classified as PS when showing DAT loss in unilateral posterior putamen, unilateral putamen, bilateral putamen, or putamen and caudate nuclei. It was defined as non-presynaptic PS (non-PS) when showing no significant DAT loss in the striatum or focal DAT loss in areas other than the posterior putamen (Fig. 1).

### Quantitative analysis of ^18^F-FP-CIT PET/CT

Image processing was performed with Statistical Parametric Mapping (Wellcome Department of Imaging Neuroscience, Institute of Neurology, University College London, London, UK) in MATLAB R2013a for Windows (The MathWorks Inc.) and MRIcro, Version 1.40 (Chris Rorden, Columbia, SC; http://www.mccauslandcenter.sc.edu/crnl/). All reconstructed PET images were spatially normalized to Talairach space by a standard ^18^F-FP-CIT PET template^10,15,16^.

Quantitative analysis was performed as described previously^12^ based on 12 volume-of-interest (VOI) templates of bilateral striatal subregions (ventral striatum, anterior caudate, posterior caudate, anterior putamen, posterior putamen, and ventral putamen) and one template of the occipital subregion. It was adjusted manually using VOI editing software (ANTIQUE; Asan Medical Center, Seoul, Korea)^16^.

The activity level in each VOI was calculated and the specific to nonspecific binding ratio (SNBR) was defined as: [(mean standardized uptake value of the striatal subregional VOI − mean standardized uptake value of the occipital VOI)/mean standardized uptake value of the occipital VOI]. We used a normal database for comparison, as previously described^10^.

### Statistical analysis

The accuracies of baseline clinical (without ^18^F-FP-CIT PET) and ^18^F-FP-CIT PET imaging diagnoses were compared, using the final clinical diagnosis made after over two years of follow-up by movement specialist as the reference standard. This specialist meticulously monitored each patient every 3–6 months for over two years for any symptom development that deviated from established diagnostic criteria.The baseline clinical diagnosis was classified as PS, non-PS, or inconclusive. The baseline ^18^F-FP-CIT PET and final clinical diagnoses were classified as PS or non-PS. Sensitivity, specificity, positive predictive value, negative predictive value, and accuracy were calculated using IBM SPSS Statistics for Windows, Version 21.0 (IBM Corp., Armonk, NY, USA).

### Ethics approval

All procedures performed in studies involving human participants were in accordance with the ethical standards of the Institutional Review Board (IRB) of Asan Medical Center and with the principles of the1964 Declaration of Helsinki and its later amendments.

## Results

### Demographic and clinical characteristics

Among the 272 patients who underwent ^18^F-FP-CIT PET imaging at the first visit, 111 had CUPS (age, 62.6 ± 10.5 y; 45 male and 66 female patients; symptom duration, 13.1 ± 8.8 months). Their demographic and clinical characteristics are presented in Table 1. The most common reasons for classifying as CUPS were showing only one of the three cardinal signs of Parkinsonism (*n* = 68, 61.2%), followed by atypical signs (*n* = 36, 32.4%) including postural tremor rather than rest tremor (*n* = 18, 16.2%) and mild rigidity (*n* = 15, 13.5%).

**Table 1:** Demographic and clinical characteristics of patients with clinically uncertain parkinsonian syndrome.

Characteristic	Value
Patients (n)	111
Age (y ± SD)	62.6 ± 10.5
Sex (M:F)	45:66
Symptom duration (mo)	13.1 ± 8.8
Follow-up duration (mo)	43.0 ± 12.4
Reason for diagnostic uncertainty at baseline	
One of the three cardinal signs of Parkinsonism	68
Two signs without bradykinesia	7
Atypical signs	
Postural rather than rest tremor	18
Mild rigidity	15
Others	3
Poor response to l-DOPA	0
Lack of disease progression	0

*SD* standard deviation, *M* male, *F* female.

### Initial clinical diagnosis and visual ^18^F-FP-CIT PET image interpretation

Among the 111 patients with CUPS, 45 (40.5%) were classified clinically as having PS, 24 (21.6%) as having non-PS, and 42 (37.8%) with inconclusive diagnosis (Table 2). Among the 45 patients classified clinically as having PS, 43 (95.6%) were classified as having PS via ^18^F-FP-CIT PET and two (4.4%) as having non-PS. The parallel values for the 24 patients clinically classified as having non-PS were 8 (33.3%) and 16 (66.7%) and were 27 (70.3%) and 15 (29.7%) for the 42 patients with inconclusive diagnosis at baseline.

**Table 2:** Classification of baseline clinical diagnosis and visual ^18^F-FP-CIT PET interpretation of patients with clinically uncertain parkinsonian syndromes (CUPS).

Baseline clinical diagnosis (*n* = 111)		Visual classification of ^18^F**-**FP-CIT PET		
		PS	Non-PS	Total
PS	*n*	43	2	45
	%	95.6%	4.4%	100.0%
Non-PS	*n*	8	16	24
	%	33.3%	66.7%	100.0%
Inconclusive	*n*	27	15	42
	%	70.3%	29.7%	100.0%

*PS* presynaptic parkinsonian syndrome, *Non-PS* non-presynaptic parkinsonian syndrome.

### Comparison of baseline clinical diagnosis and visual ^18^F-FP-CIT PET interpretation to the final clinical diagnosis

**Figure 2:**
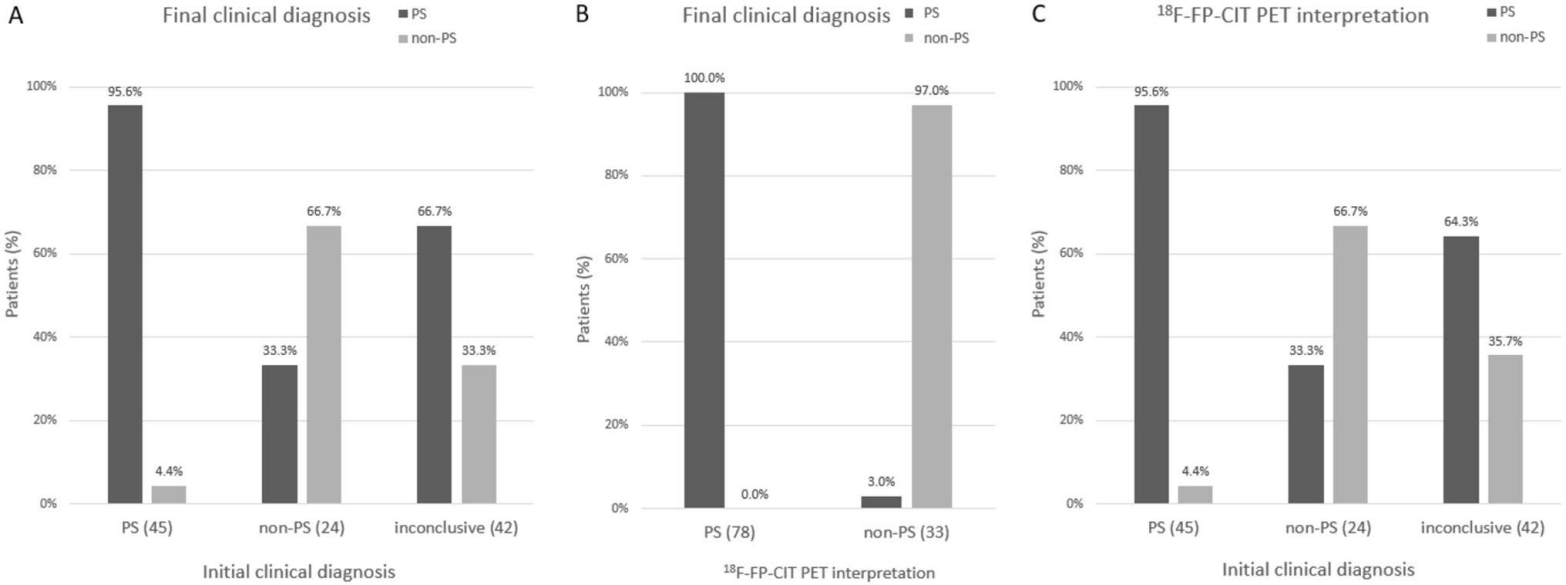
Comparison of the (**A**) initial clinical diagnosis and (**B**) visual ^18^F-FP-CIT PET interpretation to the final clinical diagnosis in patients with presynaptic Parkinsonian syndrome. Comparison of the initial clinical diagnosis to the visual ^18^F-FP-CIT PET interpretation (**C**). *PS* presynaptic Parkinsonian syndrome, *Non-PS* non-presynaptic Parkinsonian syndrome.

The final clinical diagnosis was PS for 43/45 patients classified as having PS based on the baseline clinical diagnosis, 8/24 classified as having non-PS, and 28/42 whose diagnosis was inconclusive. Among patients in whom initial clinical diagnosis (PS or non-PS) was possible, when compared to the final diagnosis, the sensitivity, specificity, accuracy, positive predictive value, and negative predictive value for baseline clinical diagnosis were 54.4, 50.0, 53.2, 95.6, and 66.7%, respectively (Fig. 2 and Table 3). Among those with initially inconclusive diagnosis, 64.2% were eventually diagnosed with PS while 35.7% were diagnosed with non-PS.

**Table 3:** Comparison of the baseline clinical diagnosis and visual ^18^F-FP-CIT PET interpretation to the final clinical diagnosis.

	Baseline clinical diagnosis	^18^F**-**FP-CIT PET
*n*	69*	111
Sensitivity (%)	54.4	98.7
Specificity (%)	50.0	100.0
Accuracy (%)	53.2	99.1
Positive predictive value (%)	95.6	100.0
Negative predictive value (%)	66.7	97.0

*Among the 111 patients with clinically uncertain Parkinsonian syndromes, 69 were classified as either presynaptic Parkinsonian syndrome or non-presynaptic Parkinsonian syndrome. The remaining 42 patients were considered inconclusive at baseline and excluded for calculating diagnostic accuracy.

All patients classified as having non-PS via ^18^F-FP-CIT PET were also classified as having non-PS based on the final clinical diagnosis. Among 33 patients classified as having PS using the initial clinical diagnosis, one was classified as having PS and 32 as having non-PS based on the final clinical diagnosis. ^18^F-FP-CIT PET diagnosis had a sensitivity of 98.7%, a specificity of 100.0%, a positive predictive value of 100.0%, a negative predictive value of 97.0%, and an accuracy of 99.1%. One patient (0.9%) received a false-negative diagnosis, and none received a false-positive diagnosis. The scans of the 76-year-old male patient with a false-negative diagnosis showed no evidence of dopaminergic deficit. The patient started showing gait disturbance with mild rigidity and bradykinesia one year before the examination. The patient’s tremor was postural rather than at rest. Initially, the patient was considered to have vascular Parkinsonism due to a mild periventricular leukoaraiosis in the white matter observed using brain magnetic resonance imaging. While the ^18^F-FP-CIT PET imaging was normal, the patient’s Parkinsonism progressed during follow-up. Therefore, this patient was considered to present scans without evidence of dopaminergic deficit (SWEDD).

### Quantitative analysis of ^18^F-FP-CIT PET/CT images

**Figure 3:**
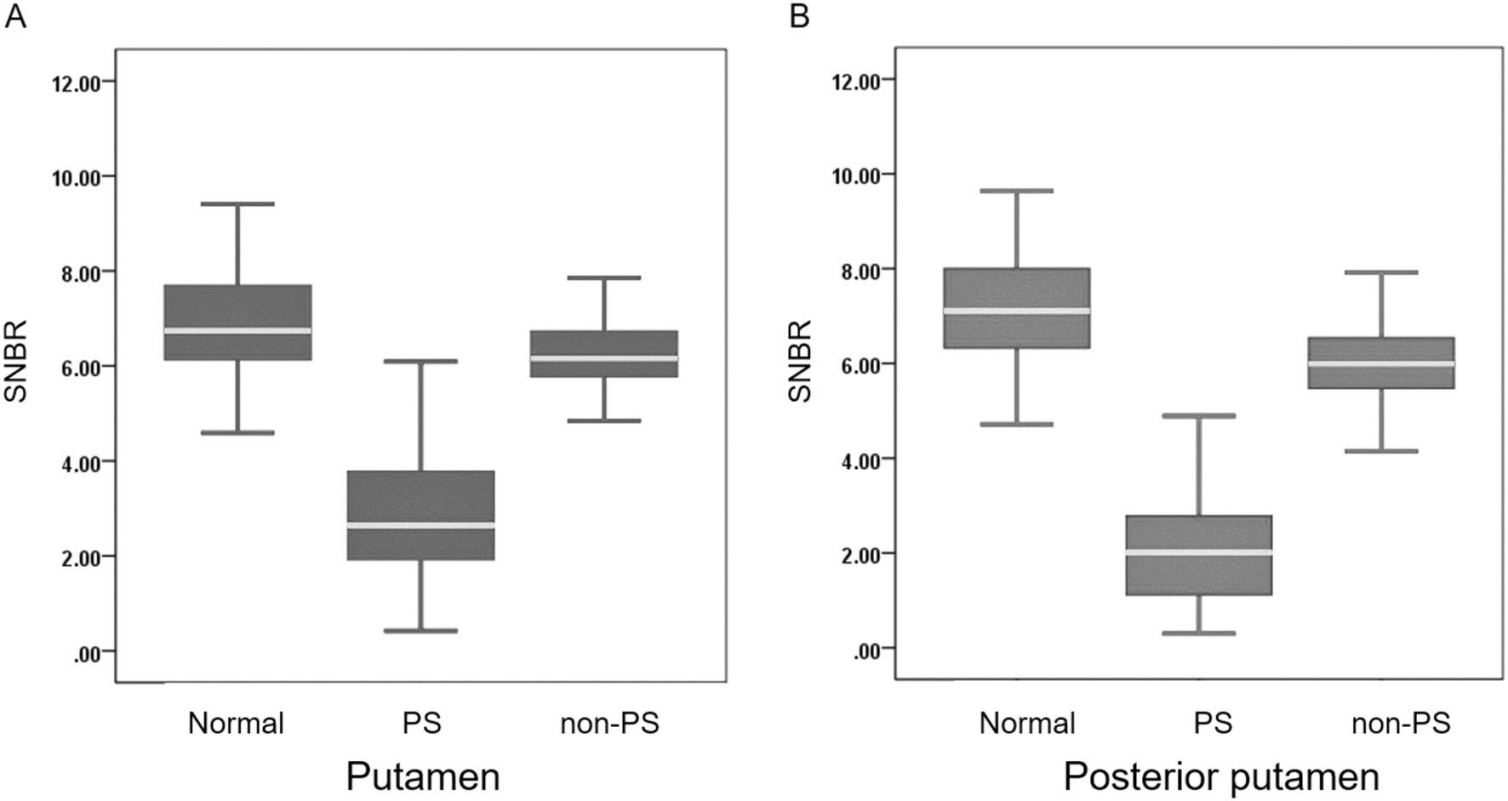
Quantitative analysis of the putamen (**A**) and posterior putamen (**B**) of subjects with normal, presynaptic Parkinsonian syndrome, and non-presynaptic Parkinsonian syndrome. *PS* presynaptic Parkinsonian syndrome, *Non-PS* non-presynaptic Parkinsonian syndrome, *SNBR* specific to nonspecific binding ratio.

The putamen SNBR for healthy controls (6.91 ± 1.32) was larger than that for patients with PS (2.78 ± 1.20; *p* < 0.001) and non-PS (6.21 ± 1.09; *p* = 0.027) based on visual ^18^F-FP-CIT PET image interpretations. The posterior putamen SNBR for healthy controls (7.26 ± 1.31) was larger than that for patients with PS (2.06 ± 1.10; *p* < 0.001) and non-PS (6.00 ± 1.07; *p* < 0.001) (Fig. 3).

## Discussion

This study investigated the diagnostic accuracy of baseline clinical ^18^F-FP-CIT PET image assessments in patients with CUPS, showing a higher accuracy for ^18^F-FP-CIT PET in differentiating PS from non-PS and some SWEDDs. Therefore, ^18^F-FP-CIT PET can be useful in reducing the rates of false-negative and inconclusive clinical diagnoses at baseline in patients with CUPS.

IPD diagnosis is still largely based on identifying its clinical features correctly. A correct diagnosis depends upon the clinical interpretation of the characteristic asymmetrical Parkinsonism responsive to anti-Parkinson therapy^17^. The clinical diagnosis of Parkinsonism is relatively straightforward in many patients^18^. However, diagnosis is challenging in cases with CUPS because the characteristic symptoms of IPD are unclear, resulting in a high portion of clinically inconclusive diagnoses^13^ and, according to a European multicenter study with repeat DAT SPECT, over-diagnoses^17^ at baseline. The diagnosis of approximately one-third of the patient with CUPS in this study was considered inconclusive at the initial clinical diagnosis stage. A prospective study supported some of the clinical difficulties in accurately diagnosing the underlying pathology in the early cases of Parkinsonian syndrome^19^. Recent clinicopathologic studies have shown that the diagnostic accuracy of IPD remains relatively low and one fourth of diagnoses are incorrect despite improvements in the diagnostic methods and the development of diagnostic clinical criteria^20^.

DAT SPECT is a widely used, cost-effective imaging technique that helps differentiate non-PS, such as ET, from PSs related to PD, multiple system atrophy, and progressive supranuclear palsy^6,21,22^. A previous study reported that the initial diagnosis was changed in 54% of patients with CUPS after DAT SPECT^13^. Among the initial clinical diagnoses in this study, two of the 45 patients initially classified as having PS were reclassified as having non-PS (4.4%), eight of the 24 patients classified as having non-PS were reclassified as having PS (33.3%), and the 42 patients classified as having inconclusive diagnosis were reclassified as having PS (*n* = 27, 64.2%) or non-PS (*n* = 15, 35.7%). Overall, the initial clinical diagnosis was changed after ^18^F-FP-CIT PET in 47% of the patients (*n* = 52). Both DAT SPECT and ^18^F-FP-CIT PET results had a higher agreement at the final diagnosis than at the baseline clinical diagnosis.

The higher proportion of SWEDDs in previous studies puts in doubt the diagnostic accuracy of DAT SPECT. The term SWEDD refers to the absence of an imaging abnormality in patients clinically presumed to have IPD^23^. The SWEDD frequency in some drug trials and imaging studies on IPD ranged between 3.6 and 19.6%^24–27^. A SWEDD rate of about 16% was reported in the Parkinson's Progression Markers Initiative (PPMI), showing similar visual and quantitative characteristics to those of healthy controls^28^. Researchers disagreed on whether SWEDD suggested different PD lookalike disorders or a benign subtype of PD; however, several longitudinal studies suggested that patients with SWEDD do not have early PD and show minimal clinical or imaging evidence of PD progression^29–31^.

Several longitudinal studies suggested a ceiling effect in DAT SPECT imaging, as the change rate shows little difference over time^32^. These patients initially showed low DAT levels in the nigrostriatal pathways, which further decreased over time, reaching a level at which Parkinsonism was detectable. Thus, statistical “floor” and “ceiling” DAT binding effects must be considered when employing imaging as an outcome measure in clinical trials on IPD. These results cast doubt on the sensitivity of DAT SPECT in detecting early-stage IPD.

Only 0.9% of the patients in this ^18^F-FP-CIT PET study presented with SWEDD after a 24-month follow-up, suggesting the suitability of ^18^F-FP-CIT PET as a biomarker for early IPD detection and disease monitoring^33,34^. A previous head-to-head comparative study of ^18^F-FP-CIT PET and ^123^I-FP-CIT SPECT found no difference between the methods in visual diagnostic accuracy^35^. Both showed high accuracy in differentiating between Parkinsonism and ET, with a sensitivity of 95–97% and 100% and a specificity of 93–100% and 97% in DAT SPECT and ^18^F-FP-CIT PET, respectively^36,37^. However, a semi-quantitative analysis indicated that ^18^F-FP-CIT PET had better contrast than ^123^I-FP-CIT SPECT^35^. This finding suggested that ^18^F-FP-CIT PET could help make more accurate decisions in equivocal cases than ^123^F-FP-CIT SPECT.

The lack of a definitive post-mortem diagnostic validation is a limitation of this study. Therefore, a follow-up period of 24 months had to be used to confirm the diagnosis. A quantitative analysis would be needed to support our results.

In conclusion, ^18^F-FP-CIT PET imaging was more accurate than a clinical diagnosis in distinguishing PS from non-PS, with a low false-negative rate. Therefore, ^18^F-FP-CIT PET imaging would be useful in reducing the false-negative and inconclusive clinical diagnosis rates at baseline in patients with CUPS.

## Data Availability

The datasets generated during and/or analysed during the current study are available from the corresponding author on reasonable request.
